# Tumour Compressing the Right Side of the Medulla Oblongata and the Nerves Proceeding from It

**Published:** 1851-11

**Authors:** 


*Tumour compressing the right side of the Medulla Oblongata and the nerves proceeding from it.*—This case was related to the Society of Biology in Paris, on March 15th, by M. Charcot.

Case.—The patient, a woman aged 48, was admitted into the Charity Hospital on February 28. She had been ill three months; and at first was under the care of M. Gubler. He was called to her a fortnight after the appearance of violent pain in the occipito-temporal region. The neck was slightly tender on pressure; the organs of sense were very susceptible; and every movement of the body brought on attempts to vomit, sometimes followed by a discharge. The pupil was much contracted; and there was obstinate constipation; but no distension or tenderness of the abdomen. The tongue was rather white, but moist; the pulse was not frequent; the skin was not hot. There was no pain in the trunk or limbs, nor paralysis, vertigo, or convulsions. Mr. Gubler diagnosed a superficial circumscribed softening of the grey matter, or rather an intracranial tumour. He applied leeches behind each ear, gave a calomel purgative, and ordered chloroform to be applied locally to calm the cephalalgia. Considerable relief ensued: but the patient was removed by her son two days after. She soon began to manifest great hebetude, and weakness in all her limbs, without pain; and at last her speech became very remarkably impaired. The cephalalgia soon became as severe as before. A seton was applied to the neck, and blisters to the thighs, but without effect; and she was admitted into hospital.

On admission, she answered questions pretty readily, but soon shewed how much her intellect was weakened and perverted. She complained of very severe pains in the cerebellar region: the head was constantly turned towards the right shoulder, but there was no rigidity or contraction of the muscles of the neck. All her limbs were relaxed; but she moved her legs and arms without much difficulty, when desired to do so. When pinched, she felt it readily. There was no strabismus, ptosis, or deafness. The pupils were equally dilated. She had never vertigo, muscæ volitantes, or noises in the ears. There were no signs of paralysis of the facial nerve. She protruded her tongue readily; but the point was immediately carried to the right. She could, however, easily turn it in her mouth, and could turn the point to the left when desired to do so. The movements of deglutition were perfect. The vomiting and constipation continued as long as the patient was in the hospital. A kind of coma supervened, and the patient died on March 9th: she never had convulsions.

From the symptoms it seemed probable that there was a tumor compressing the ninth nerve; but there were no symptoms of syphilis, tubercles or cancer. On post-mortem examination, a tumor, of the size and shape of a large pigeon's egg, was found on the front of the right lobe of the cerebellum, where it had formed a hollow. It also compressed the right peduncle of the brain, and pushed the medulla oblongata towards the left side. It had flattened the auditory, facial, pneumogastric, glosso-pharyngeal, and especially the hypoglossal nerves on the right side. The fifth pair was also much flattened. The third and sixth nerves were untouched. The tumor was very feebly adherent, and was easily removed by tearing some very loose cellular tissue. It was hard and dry, and grated under the scalpel: it was somewhat mammillated. Examined with the microscope, it presented fibro-plastic tissue, with a predominance of the fibrous tissue.—*Lond. Journ. of Med.*

